# Predictors of Visceral Leishmaniasis Relapse in HIV-Infected Patients: A Systematic Review

**DOI:** 10.1371/journal.pntd.0001153

**Published:** 2011-06-07

**Authors:** Gláucia F. Cota, Marcos R. de Sousa, Ana Rabello

**Affiliations:** 1 Post-Graduate Program in Health Sciences, René Rachou Institute, Fundação Oswaldo Cruz, Belo Horizonte, Minas Gerais, Brazil; 2 Post-Graduate Program in Adult Health Sciences, Federal University of Minas Gerais, Belo Horizonte, Minas Gerais, Brazil; 3 Eduardo de Menezes Hospital, Fundação Hospitalar do Estado de Minas Gerais-FHEMIG, Belo Horizonte, Minas Gerais, Brazil; Hebrew University-Hadassah Medical School, Israel

## Abstract

**Background and Objectives:**

Visceral leishmaniasis (VL) is a common complication in AIDS patients living in *Leishmania*-endemic areas. Although antiretroviral therapy has changed the clinical course of HIV infection and its associated illnesses, the prevention of VL relapses remains a challenge for the care of HIV and *Leishmania* co-infected patients. This work is a systematic review of previous studies that have described predictors of VL relapse in HIV-infected patients.

**Review Methods:**

We searched the electronic databases of MEDLINE, LILACS, and the Cochrane Central Register of Controlled Trials. Studies were selected if they included HIV-infected individuals with a VL diagnosis and patient follow-up after the leishmaniasis treatment with an analysis of the clearly defined outcome of prediction of relapse.

**Results:**

Eighteen out 178 studies satisfied the specified inclusion criteria. Most patients were males between 30 and 40 years of age, and HIV transmission was primarily via intravenous drug use. Previous VL episodes were identified as risk factors for relapse in 3 studies. Two studies found that baseline CD4+ T cell count above 100 cells/mL was associated with a decreased relapse rate. The observation of an increase in CD4+ T cells at patient follow-up was associated with protection from relapse in 5 of 7 studies. Meta-analysis of all studies assessing secondary prophylaxis showed significant reduction of VL relapse rate following prophylaxis. None of the five observational studies evaluating the impact of highly active antiretroviral therapy use found a reduction in the risk of VL relapse upon patient follow-up.

**Conclusion:**

Some predictors of VL relapse could be identified: a) the absence of an increase in CD4+ cells at follow-up; b) lack of secondary prophylaxis; and c) previous history of VL relapse. CD4+ counts below 100 cells/mL at the time of primary VL diagnosis may also be a predictive factor for VL relapse.

## Author Summary

Visceral leishmaniasis (VL) is the most serious form of an insect-transmitted parasitic disease prevalent in 70 countries. The disease is caused by species of the *L. donovani* complex found in different geographical regions. These parasites have substantially different clinical, drug susceptibility and epidemiological characteristics. According to data from the World Health Organization, the areas where HIV-*Leishmania* co-infection is distributed are extensive. HIV infection increases the risk of developing VL, reduces the likelihood of a therapeutic response, and greatly increases the probability of relapse. A better understanding of the factors promoting relapses is essential; therefore we performed a systematic review of articles involving all articles assessing the predictors of VL relapse in HIV-infected individuals older than 14 years of age. Out of 178 relevant articles, 18 met the inclusion criteria and in total, data from 1017 patients were analyzed. We identified previous episodes of VL relapse, CD4+ lymphocyte count fewer than 100 cells/mL at VL diagnosis, and the absence of an increase in CD4+ counts at follow-up as major factors associated with VL relapse. Knowledge of relapse predictors can help to identify patients with different degrees of risk, facilitate and direct prophylaxis choices, and aid in patient counseling.

## Introduction

Visceral leishmaniasis (VL) and human immunodeficiency virus (HIV) co-infection has emerged as a serious disease pattern [1], [2]. HIV infection increases the risk of developing VL by 100 to 2,320 times in endemic areas [3], [4] and, on the other hand, VL promotes the clinical progression of HIV disease and the development of AIDS-defining conditions [5]. Both infections switch the predominantly cellular immunological response from Th1 to Th2 through complex cytokine mediated mechanisms leading to a synergistic detrimental effect on the cellular immune response [6], [7], [8]. Other important findings related to HIV-*Leishmania* co-infection is a reduction in therapeutic response and high rate of relapse, which is the clinical deterioration after clinical improvement, observed in 25–61% of patients [9], [10], [11], [12]. Although the term recurrence has also been used as synonym for relapse, recurrence applies to the finding of a parasite repeatedly. It is important to emphasize that neither of these two terms distinguishes parasitological persistence from re-infection.

The poor therapeutic outcome, the high rate of relapse, the poliparasitic nature of VL in HIV-infected persons, as well as the atypical manifestations of the disease and the impaired access to health-care resources make HIV-infected individuals prone to enlarge the number of human reservoirs [13]. This concern is of utmost importance in Asia, where HIV and *Leishmania* co-infections are increasingly being reported in countries that are also facing parasite resistance to antimonial drugs [14].

Recent changes in the epidemiological patterns of HIV and *Leishmania* infections are likely to lead to a greater degree of overlap and greater risk of co-infection and they justify increased alertness. From a global epidemiologic viewpoint, two parallel trends are alarming: the ruralization of the HIV pandemic and the urbanization and spread of VL [1], [15]. World Health Organization (WHO) [16] reports that the public health impact of leishmaniasis worldwide has been grossly underestimated for many years because notification was compulsory in only 32 of the 88 countries where 350 million people were at risk. The reported global incidence of co-infection is likely underestimated either because VL has not been included in the list of AIDS related opportunistic infection in all endemic areas.

Before the widespread use of antiretroviral therapy, such co-infection was common in Europe [5]. The co-infection is now becoming proportionately more prominent in areas with poor access to antiretrovirals, such as Africa. In areas where it is available, highly active antiretroviral therapy (HAART) has changed the course of the HIV/AIDS epidemic and the outcome of associated opportunistic infections. However, evidence of relapse rate reduction in patients using HAART is conflicting [17]. This work is a systematic review of studies describing the predictors of VL relapse in HIV-infected patients.

## Methods

### Search Strategy and Selection Criteria

**Figure 1:**
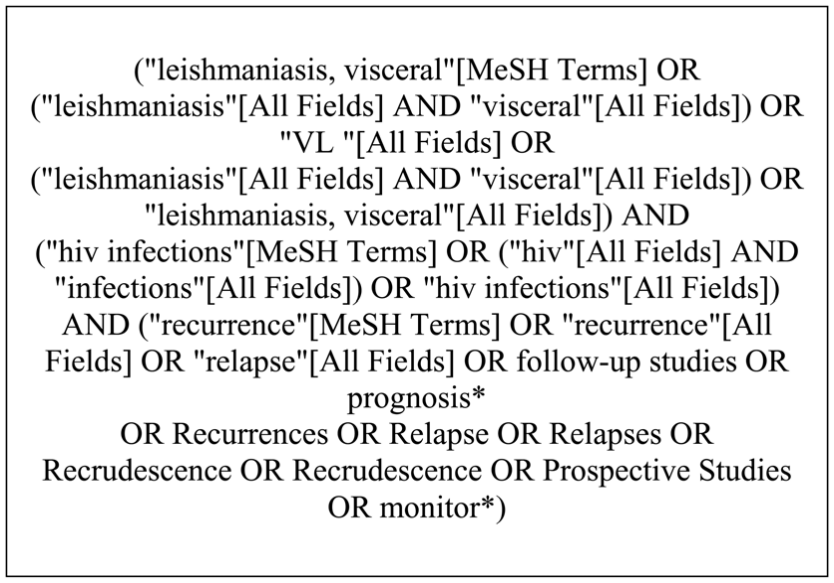
Terms used in Medline/PubMed search.

This review was conducted on all papers published before July, 31, 2010. To ensure scientific rigour, the Preferred Reporting of Systematic Reviews and Meta-Analysis (PRISMA) guidelines [18] were used for systematic data synthesis. Studies were identified by a Medline/PubMed search using a combination of terms that has been shown to maximize sensitivity [19]. The search terms used are shown in Figure 1. The LILACS and Cochrane databases were used for literature review using a Boolean combination of search terms. Additional reports were located using a manual search of references from retrieved papers. Two independent reviewers (GFC and MRS) initially checked the lists of titles and abstracts identified by this search to determine whether an article contained relevant data. Studies were considered eligible if they were presented in an original article, examined HIV-infected individuals over 14 years of age with a VL diagnosis, included follow-up after the leishmaniasis treatment and clearly analyzed predictors of relapse.

There were no restrictions on the publication language, date of publication, use of secondary prophylaxis, or duration of follow-up in the study. We excluded studies evaluating fewer than ten cases and studies evaluating mixed populations of HIV-infected and uninfected subjects unless separated results for HIV patients were identified. The selected articles were read in full to confirm eligibility.

Data were extracted directly from the full-length articles into structured tables containing all of the descriptive variables and relevant outcomes. The following information was extracted: country and period of enrollment; sample size; clinical characteristics of the included patients; study design; the number of excluded patients if specified; statistical analyses utilized; duration of follow-up and number of subjects lost to follow-up; outcome of interest; prognostic variables assessed in each study and quality of the regression model [20], [21], [22]. When data were available tests required for completion of the tables were performed. To summarize the results regarding secondary prophylaxis, the software Comprehensive Meta-Analysis Version 2.2.048 was used.

## Results

**Figure 2:**
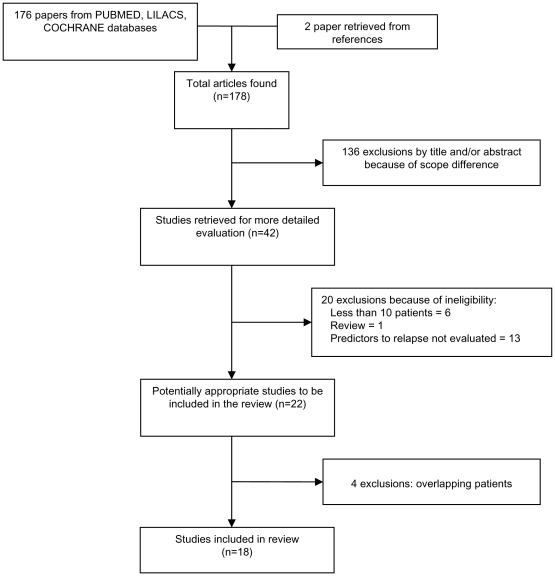
Study selection process.

Our selection process is illustrated in Figure 2. Of 178 articles, 136 were excluded because they did not meet the inclusion criteria following reading of titles and/or abstracts. Twenty more articles were excluded after reading the entire article: six analyzed less than ten patients [23], [24], [25], [26], [27], [28], [29], one was a review [30], and thirteen did not evaluate the risk on relapse of different predictors [3], [28], [31], [32], [33], [34], [35], [36], [37], [38], [39], [40], [41]. Four studies [42], [43], [44], [45] were excluded because they included cases published elsewhere [10], [46], [47]. Thus, 18 studies (Table S1) satisfied the specified inclusion and exclusion criteria and constituted the basis of this investigation.

### Studies and Patients

Table S1 summarizes the characteristics of the 1017 patients encompassed by the 18 included studies. The year of study publication ranged from 1989 to 2008. The design of 8 of the studies examined was prospective. Fourteen studies were reported in Spain, two in Italy, and one in Ethiopia and one in France. Eight studies had an enrollment period exclusively after 1996, when HAART became available. Twelve papers stated the proportions of patients receiving HAART involving two nucleoside reverse transcriptase inhibitors and one or two protease inhibitors or non-nucleosides reverse transcriptase inhibitors at VL diagnosis or at relapse or both.

A large proportion of the patients in these studies (87.7%) were male and most were young adults; the median or mean ages reported varied from 27 to 37 years (Table S2). In the 14 studies in which patients' presumed transmission route was known, 72.3% (420/581) of the infections were likely due to intravenous drug use. The median CD4+ T lymphocyte count ranged from 11 to 82 cells/mL. Most patients had an AIDS-defining condition [48] at the time of VL diagnosis (332/572, 58% of patients).

In the majority of the studies, the diagnosis of VL was established by direct demonstration of amastigotes (by cytological study of Wright stains) or by the observation of promastigote growth in samples cultured in specific media. In one study [49], the VL diagnosis was supported either by positive results from *Leishmania*-specific PCR (polymerase chain reaction) of peripheral blood or bone marrow samples. Three studies [47], [50], [51] also included patients diagnosed by serologic tests (direct agglutination, indirect immunofluorescence or rK-39 dipsticks).

The drug used in the treatment of the primary episode of VL was reported for 89% of the treated patients. Of this total, 73.4% of cases (733 patients) were treated with pentavalent antimonial drugs, 12.4% with amphotericin B deoxycholate (124 patients), and 2.1% (21 patients) received amphotericin in lipid formulations. A minority of patients (1.2%) received pentamidine isethionate and three papers included patients treated with miltefosine [47] or unconventional regimens such as a combination of allopurinol with an azole compound [50], [52]. A test of cure (staining with Giemsa stain and parasite culture or PCR) at the end of treatment was carried out in 8 of 18 studies. In most of these studies, this control was performed for patients whose clinical response was uncertain. Secondary prophylaxis for leishmaniasis was reported in eleven studies.

Three studies explored the impact of mono or dual antiretroviral therapy at VL diagnosis [47] or during the follow-up [50], [53] on relapse. Only one [47] of these studies demonstrated a reduction in relapse rate compared with patients who did not undergo retroviral therapy. Similarly, only one [49] of four studies [10], [49], [51], [54] that followed patients on HAART at VL diagnosis reported a reduction in relapse rate. HAART use on follow-up has also been studied in relation to risk of relapse and none of the five [9], [51], [52], [54], [55] studies showed reduction on VL relapse rate.

**Figure 3:**
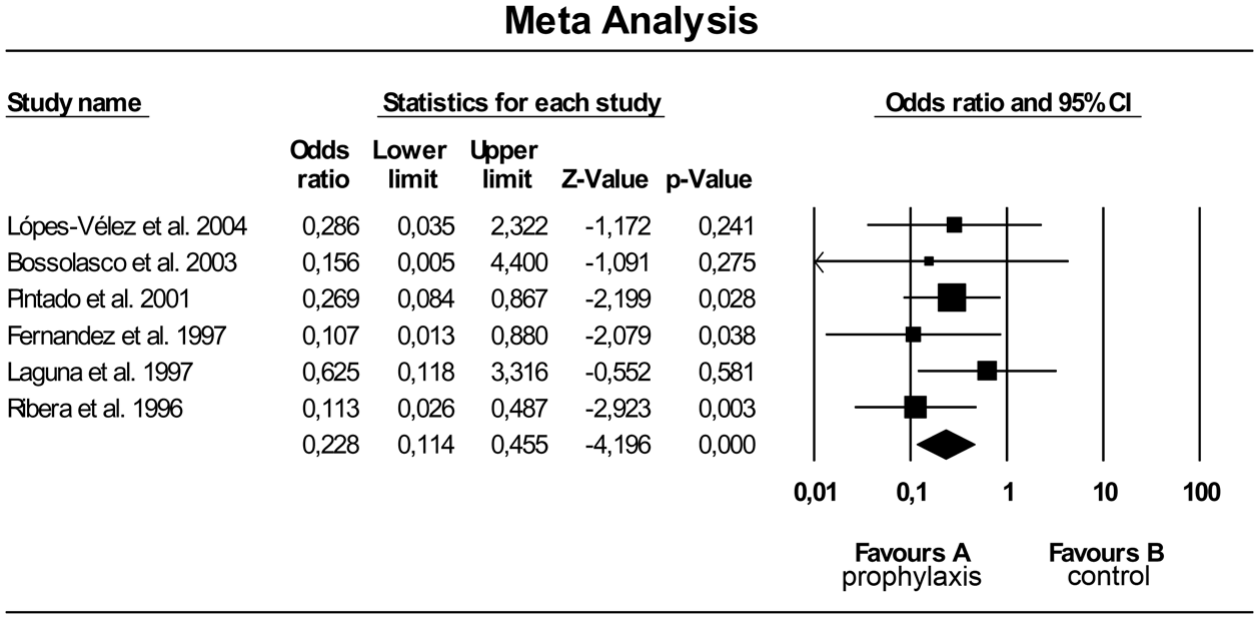
Meta-analysis of secondary prophylaxis results. Footnote: I^2^ = 0% Egger test for publication bias was negative, p = 0.76.

Two studies [52], [54] that evaluated VL prophylaxis without specifying the drug used noted a significant reduction in relapse. In a report of ten cases, Bossolasco et al. [55] showed that the relapse rate in patients groups with and without prophylaxis were 60% and 100%, respectively, but this difference did not reach statistical significance. Three studies evaluated specific prophylactic regimens (antimony compounds [46], [50] and liposomal amphotericin [50]) and demonstrated reduction on VL relapse. Although the confidence intervals did not reach statistical significance, another author [56] concluded that lipid-complexed amphotericin prophylaxis also reduced the relapse rate. Finally, Laguna et al. [57] showed a trend towards (p = 0,08) a reduction in VL relapse rate following treatment with pentamidine prophylaxis. A meta-analysis of results from all studies evaluating the impact of secondary prophylaxis is shown in Figure 3. This analysis could consistently demonstrate that secondary prophylaxis reduces VL relapse rate.

CD4+ lymphocyte count at VL diagnosis and follow-up has been studied in relation to risk of relapse. Nine articles [10], [11], [12], [46], [50], [51], [52], [55], [58] compared CD4+ lymphocyte cell counts at VL diagnosis between relapsing and non-relapsing patients as a continuous variable. Neither of these studies showed significant differences between these two groups. On the other hand, two studies [47], [49] that compared relapse rate between patients with CD4+ count at VL diagnosis as a dichotomic variable (above and below than 100 cell/mL) noted that the arms with higher CD4+ counts had lower relapse rate. Similarly, an increase in CD4+ lymphocyte count at follow-up was protective against VL relapse in 5 of 7 studies [10], [11], [49], [55], [58]. In another study [12], univariate analyses of CD4+ counts at follow-up revealed a trend towards a reduction in relapse (p = 0.09).

Other variables explored in relation to relapse are shown in Table S3. Factors such as age, route of HIV transmission, history of intravenous drug use, HIV viral load at VL diagnosis, various clinical findings, specific anti-*Leishmania* treatments given, time from VL diagnosis to the introduction of protease inhibitor therapy, HAART compliance, the presence of an AIDS-defining disease before VL diagnosis and the presence of serum anti-*Leishmania* antibodies were not substantially different between relapsing and non-relapsing patients. Tuberculosis co-infection [47], hepatitis C virus co-infection [49] and an incomplete course of VL treatment [52] were evaluated in multivariate analysis and showed a statistically significant association of these conditions with the occurrence of relapse. Previous VL episodes were identified as risk factors for relapse in 3 studies, two of which were multivariate analyses.

### Prognostic Variables and Statistical Analysis

The statistical quality and the presentation of methods and results in many studies were poor. In nine studies, the Kaplan-Meier method was used in a univariate survival analysis to analyzed VL relapse. Three prospective studies and two retrospective cohort studies employed Cox regressions for multivariate analysis of independent predictors. One study randomized patients to compare prophylaxis (liposomal amphotericin versus no treatment) and performed multivariate analysis to compare relapse rates by logistic regression, including some predictors as covariates. None of these six studies mentioned collinearity assessment (i.e., a high degree of correlation between 2 predictive variables) or developed a risk score for relapse based on their multivariable results. Also, none of the multivariate analyses reported a goodness-of-fit test of their models. Other studies analyzed isolated relapse predictors by univariate association tests in series of prospective or retrospective cases or in intervention studies.

## Discussion

The present study is the first systematic review of predictors of VL relapse in HIV-infected patients. Our main conclusions are that VL relapse in HIV-infected patients receiving HAART is high and that secondary prophylaxis provides some protective effect but does not completely prevent the occurrence of relapse. We found that patients who did not relapse showed significantly higher CD4+ count at follow-up than patients with relapsing course. Our analysis also suggests that CD4+ count greater than 100 cell/mL at VL diagnosis reduces the odds of relapse. Unlike other opportunistic infections there are some reports of VL relapse in patients with a CD4+ count greater than 200 cell/mL in Ethiopia, and rarely in Europe [9]. This evidence shows that factors other than a CD4+ cell increase are involved in VL control. A threshold for safely discontinuing of secondary prophylaxis has not been established because of these uncertainties.

Most cases reported showed severe reductions in T cells. It could indicate that VL affects HIV-1 patients who exhibit a significant disturbance of cellular immunity; however, VL by itself may reduce CD4+ lymphocyte counts [59]. On the other hand, a CD4+ count greater than 100 cell/mL at VL diagnosis is a potential protective factor against relapse, although the analysis of this beneficial effect may be complicated by the immunosuppression of many the patients included in the studies. When analyzing the CD4+ count range and number of patients with CD4+ counts of greater than 100 cell/mL in the two studies [47], [49] demonstrating an association between higher baseline CD4+ counts and reduced VL relapse, it is possible to speculate that studies that did not demonstrate an influence of CD4+ cells had few patients with CD4+ counts of greater than 100 cell/mL. Studies using animal models reported that CD4+ cells are responsible for the initial control of parasite proliferation and dissemination [60]. Thus, a low initial CD4+ count might allow a wide dissemination of the parasite throughout the phagocytic mononuclear system at the beginning of infection, increasing the number of sites that could harbor quiescent parasites (so-called “sanctuaries”) [61].

Relapses of VL are suggested to occur mainly in individuals with poor responses to antiretroviral treatment who have no improvement in CD4+ counts [11], [12], [58], [62], with a few exceptions [9], [47]. The evolution of patients who acquire VL and thereafter show a significant increase in CD4+ counts while on HAART is currently receiving attention [47] [50] [51] [52]. It has already been established that the outcome of VL is not influenced by humoral immunity but appears to be regulated by CD4+ T helper cells with different patterns of cytokine activity [63]. Protective immunity can be attributed to T helper (Th)-1 cells, whereas Th-2 cell responses produce IL-4 and IL-10 and facilitate the intracellular survival of the parasite [64]. It might be expected that highly active antiretroviral drug combinations would favor an immunological shift from type 2 to type 1 cytokines in HIV-infected individuals. However, increased CD4+ values in peripheral blood and lymphoid tissues as a result of antiretroviral therapy may have negligible effects on cytokine production during the first 24 weeks [65]. In addition, patients on HAART show an initial increase in the CD4+ memory subset, whereas naive CD4+ cells consistently increase only after 1 year [66].

It is known that HIV patients who are receiving HAART have fewer opportunistic infections and recent data shows that there has been a decline in the incidence of VL after the introduction of HAART [41], [54], [67], [68], [69]. HAART seems to be insufficient to prevent VL relapse. Studies in patients receiving HAART showed a relapse rate similar to other studies performed in the pre-HAART era. Only one [49] observational study noted a reduction in the relapse rate among patients on HAART at VL diagnosis. None of the studies reported a statistically significant difference in VL relapse between patients receiving and not receiving HAART on follow-up. These disappointing results so far disagree with a statistically significant association between improvement of CD4+ count at follow-up and reduction of VL relapse. They may be due to the small sample sizes of the studies performed, poor patient adherence to antiviral therapy or insufficient immune response. One possibility to be explored in the future is the role of cytokines [70] influencing the control of VL independently of the CD4+ lymphocyte. The heterogeneity of zymodemes that exhibit different degrees of virulence or parasite burden could contribute to the differences observed in the host immune response and clinical evolution [9]. HAART increases CD4+ count thus influencing the control of VL, but may not be enough in this complex scenario created by the co-infection HIV and *Leishmania*. Fernandéz-Cotarelo et al. [54] and others [41] have shown a decrease in the number of new episodes of VL in HIV-infected patients receiving HAART but also a tendency toward VL relapse. According to these authors the high rates of relapse could be explained by the increased patient survival that results from effective antiretroviral therapy.

Previous episodes of VL were strongly associated with relapse. Also in agreement with the immune-inflammatory theory, it was hypothesized that the enhancement of the Th-2 response following one early relapse could complicate or prevent the later control of *Leishmania* infection [54].

Secondary prophylaxis seemed to only partially protect against relapse. Some of studies that observed a reduction in VL relapse following the use of secondary prophylaxis had few patients on HAART, which may not reflect the current reality. Data analysis suggests that the small sample sizes and heterogeneity of regimens used make the results less robust. Nevertheless, the evaluation of these studies through meta-analysis indicates a clear benefit of secondary prophylaxis in reducing VL relapse. Based on six studies whose data were available, the average relapse rate in patients not receiving secondary prophylaxis was 67%, while in the secondary prophylaxis arm, the relapse rate was 31%. Given this difference, the estimated total sample size needed for a study with 80% power would be 70 patients. Three out of the six studies examining secondary prophylaxis were not able to demonstrate statistical significance, possibly because of small sample sizes. It is important to emphasize that despite the heterogeneity of prophylaxis regimens used; statistical results are positively homogeneous in meta-analysis.

Thresholds for safe discontinuation of secondary prophylaxis for Spanish patients have been suggested to be CD4+ counts of 200 [71] and 350 cells/mL [11]. Differently of the European experience, one Ethiopian study [47] has shown that many patients suffering relapse (11 from 39 cases) had a CD4+ count above 200 cells/mL before relapse. These data may suggest that *L. donovani*, the predominant causative agent of VL in east Africa and south Asia, is a more virulent and anthroponotic species than *L. infantum*. Another plausible explanation for this difference may be the influence of other variables that can affect the host immune response such as nutritional status and the presence of other infections and co-morbidities.

It has been postulated that the maintenance of an undetectable viral load protects against the development of VL [17] and that a high viral load could predict a weak response to antiparasitic treatment [12] although there are contradictory reports on this point [54], [72]. None of the papers reviewed here linked HIV load by PCR at VL diagnosis with relapse. On the other hand HIV load by PCR at follow-up was statistically related to relapse in one [58] of four studies that evaluated this variable in a univariate analysis. These observations support the idea that a sustained immunological response is more important than a virological response to cure VL in HIV-infected patients.

It is important to note that a wide range of therapeutic drugs were utilized for the treatment of VL in the studies we have reviewed. There was no notable difference in the relapse rate with regard to specific VL treatment used (all analyzed in univariate analysis); however only four studies explored this association and most of them included a limited number of patients and only two [11], [73] involved randomly assigned patients. Few comparative clinical studies have been conducted of the efficacy of treatment for HIV–VL co-infection outside the Mediterranean area. In some instances [74], [75], the development of drug resistance could contribute to therapeutic failure and the relapsing course observed in HIV-infected patients. These observations do not allow us to refute the influence of anti-parasite treatment on relapse outcome.

### Study Limitations

Although we have made an extensive review, our analysis includes studies with different definitions of cure and different lengths of follow-up. Cure is seldom defined parasitologically in these studies and the difference between treatment failure and relapse is arbitrary in some studies. It is possible that some episodes of relapse in the group of patients in which parasitological cure were not documented by bone marrow examination were treatment failures rather than relapses. Moreover, re-infection was not distinguished from relapse in any paper. There is a high degree of heterogeneity in the evaluated populations as shown by the wide range of reported mortality (6.5% to 83.8%), treatment failure (0 to 47.6%) and relapse rates (20% to 70%). These studies included patients with different degrees of immunosuppression, and different treatment and prophylaxis regimens. Also, there are differences in the study designs, the types of statistical methods used and the prognostic variables included in analysis. These variations may have resulted in patient selection bias or low statistical power, thus hampering a meta-analysis of all studied predictors of relapse. In spite of these limitations, we believe that the meta-analysis results of secondary prophylaxis are consistent, considering the available evidence. In addition, the quality of published reports was heterogeneous and usually poor. Despite these limitations, this review may assist clinicians in making decisions and may also help in the design of future studies.

### Conclusion

The results of this systematic review suggest there are identifiable predictive factors of VL relapse, such as previous episodes of VL relapse and lack of recovery of CD4+ lymphocyte numbers after primary visceral leishmaniasis. HAART did not produce the anticipated decrease in the incidence of VL relapses and more data is needed in order to better assess the evolution of VL in the HAART era. In contrast, secondary prophylaxis was shown to be protective against relapse. CD4+ count below 100 cells/mL at the time of VL primary diagnosis is a potential predictor of relapse.

Based on these observations, a high-risk population might be identified and such patients might then be eligible for secondary prophylaxis. Strong surveillance will certainly contribute to improved data quality for decision-makers in this complex scenario. Randomized trials to compare the efficacy of different drugs and their role either in treatment or in prophylaxis are required.

## Supporting Information

Table S1
**Parasitological control.** Identification of *Leishmania* amastigotes by direct examination or by isolation of promastigotes in culture of tissue samples dAmB: amphotericin B deoxycholate LAmB: liposomal amphotericin LipAmB: amphotericin B lipid complex. PA: Pentavalent antimonial compounds **SD**: standard deviation **IRQ**: interquartile range **
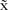
**: median **μ**: mean.(DOC)

Table S2
**VL**: Visceral leishmaniasis **Parasitological confirmation**: identification of *Leishmania* amastigotes by direct examination or by isolation of promastigotes in culture of tissue samples #**Serology confirmation**: *Leishmania* direct agglutination positive **§Biologic confirmation**: identification of *Leishmania* amastigotes by direct examination or by isolation of promastigotes in culture of tissue samples or Leishmania-specific PCR on peripheral blood/bone marrow **dAmB**: amphotericin B deoxycholate **LAmB**: liposomal amphotericin B **LipAmB**: amphotericin B lipid complex **PA**: Pentavalent antimonial compounds **Hemo**: transfusion route **IDU**: intravenous drug user **HETERO**: heterosexual contacts **HOMO**: men who have sex with men **sexual**: heterosexual or homosexual contacts **SD**: standard deviation **IRQ**: interquartile range ***♯*** if the information was available 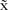: median μ: mean.(DOC)

Table S3 **Yes**: positive association **No**: negative association **VL**: Visceral leishmaniasis **HAART**: highly active antiretroviral therapy **HVC**: hepatitis C virus * multivariate analysis.
(DOC)
