# Supplementary material for: Predictors of Visceral Leishmaniasis Relapse in HIV-Infected Patients: A Systematic Review
Source: PLoS Negl Trop Dis. 2011 Jun 7;5(6):e1153. doi: 10.1371/journal.pntd.0001153 (PMC3110161; doi:10.1371/journal.pntd.0001153)
Supplement: Table S1 — Parasitological control. Identification of Leishmania amastigotes by direct examination or by isolation of promastigotes in culture of tissue samples dAmB: amphotericin B deoxycholate LAmB: liposomal amphotericin LipAmB: amphotericin B lipid complex. PA: Pentavalent antimonial compounds SD: standard deviation IRQ: interquartile range : median μ: mean. (DOC) [file pntd.0001153.s001.doc]

**Table S1. Visceral leishmaniasis relapse in HIV-1 infected patients**: characteristic of studies and outcomes

|  |  | | | | |  | | | | | |  | | |
| --- | --- | --- | --- | --- | --- | --- | --- | --- | --- | --- | --- | --- | --- | --- |
| *Reference* | | *Period of enrollment and Country* | *Sample size* | *Study design* | *Cure assessment* | | *^x̃^Median or ^μ^mean length of follow-up (range or SD) month* | *Patients lost to follow-up (%)* | *Treatment failure %* | *Mortality rate (%)* | *Secondary prophylaxis (number of patients: prophylactic regimen)* | | *Relapse rate*  *(%)* | *Statistical analysis* |
| Ter Horst, 2008 | | 2003 to 2006, Ethiopia | 161 without antiretroviral therapy | Retrospective cohort single center | Clinical improvement and parasitological control (some patients) | | **x̃** 3,1 (0-36.5) | 120 (74.7) | Not informed | 11/161 (6.8) | All patients: no prophylaxis | | Not informed | Multivariate |
|  |  |  | 195 with antiretroviral therapy |  |  |  | **x̃** 7,1 (0.5-33.5) | 37 (19.1) |  | 28/195 (14.4) |  |  | 43/195 (22) |  |
| Bourgeois, 2008 | | 1995 to 2004, France | 27 | Prospective observational two centers | Not informed | | **x̃** 51 (5-108) | 2 (7.4) | Not informed | 7/27 (26) | All patients: dAmB 0.6 to 0.7 mg/kg or LAmB 3-4mg/kg twice monthly or monthly | | 16/27 (59.3) | Multivariate |
| Molina, 2007 | | 2001 to 2005, Spain | 15 | Prospective non-controlled single center | Clinical improvement and parasitological control (some patients) | | **x̃** 14 (5-44) | 2/15 (13.3) | 0 | 1/15 (6.6) | All patients: LAmB 5mg/kg every 3 weeks (replaced by miltefosine after 2003 in three patients) | | 3/15 (20) | Univariate |
| Pasquau, 2005 | | 1988 to 2001,  Spain | 155 | Retrospective observational multicenter | Clinical improvement | | **x̃** 8.4 (IQR 1,8-19.4) | Not informed | 20% | 32/155 (21) | 34 patients: no prophylaxis | | 37/96 (38.5) | Multivariate |
|  |  |  |  |  |  |  |  |  |  |  | 30 patients: PA; 2 patients: dAmB; 20 patients: pentamidine; 4 patients: LAmB; 6patients: others agents | |  |  |
| Mira, 2004 | | 1989 to 2002, Spain | 21 without secondary prophylaxis | Retrospective cohort two centre | Not informed | | **x̃** 25 (2−61) | 4 (12.9) | Not informed | 10/31 (32.2) | 21 patients: no prophylaxis | | 8/21 (38) | Univariate |
|  |  |  | 10 with secondary prophylaxis |  |  |  | **x̃** 30 (4−53) |  |  |  | 10 patients: secondary prophylaxis with drug not informed | | 1/10 (10) |  |
| López-Vélez, 2004 | | 1997 to 1999, Spain | 17 | Prospective comparative multicenter randomized for prophylaxis use comparison | Clinical improvement and parasitological control | | **x̃** 12 | Not informed | 0 | Not informed | 9 patients: no prophylaxis | | 4/8 (50) | Multivariate |
|  |  |  |  |  |  |  |  |  |  |  | 8 patients: LipAmB 3mg/kg/d every 3 weeks | | 7/9 (88.8) |  |
| Fernandéz-Cotarelo, 2003 | | 1994 to 2000, Spain | 34 | Retrospective observational single center | Not informed | | Not informed | Not informed | Not informed | Not informed | Number of patients under secondary prophylaxis not informed. | | 13/34 (38.2) | Univariate |
| Bossolasco, 2003 | | Not informed, Italy | 10 | Prospective observational two centers | Clinical improvement | | **x̃** 8,9 (1.5-60) | 1/10 (10) | Not informed | 0 | 4 patients: no prophylaxis | | 4/4 (100) | Univariate |
|  |  |  |  |  |  |  |  |  |  |  | 5 patients: prophylaxis with LAmB 3mg/kg once a month | | 3/5 (60) |  |
| Casado, 2001 | | 1996 to 1997, Spain | 10 | Prospective observational single center | Not informed | | **x̃** 31 | Not informed | Not informed | Not informed | All patients: : no prophylaxis | | 7/10 (70) | Univariate |
| Pizzuto, 2001 | | 1997 to 1999, Italy | 10 | Prospective observational multicenter | Not informed | | **x̃** 22 (7.5-27.5) | Not informed | Not informed | 3/10 (30) | All patients: : no prophylaxis | | 6/10 (60) | Univariate |
| Pintado, 2001 | | 1974 to 1997, Spain | 80 | Retrospective observational single center | Clinical improvement and parasitological control (some patients) | | **μ** 13.8 (3–44) | 16/80 (20) | 23.3 | 43/80 (53.7) | 33 patients: no prophylaxis  9 patients: LAmB; 7 patients: PA; 3 patients: allopurinol, 1 patient: pentamidine | | 22/33 (66.7) | Multivariate |
|  |  |  |  |  |  |  |  |  |  |  |  |  | 7/20 (35) |  |
| Berenguer, 2000 | | 1998 to 2000, Spain | 15 | Prospective observational single center | Not informed | | **x̃** 15.8 (1-22) | 0 | Not informed | Not informed | All patients: : no prophylaxis | | 3/15 (20) | Univariate |
| Villanueva, 2000 | | 1996 to 1999, Spain | 32 | Prospective observational single center | Clinical improvement and parasitological control | | **μ** 14,7 (3-34) | 10/32 (31) | 6.3 | 3/32 (9.4) | All patients: no prophylaxis | | 5/20 (25) | Univariate |
| Laguna, 1999 | | 1994 to 1996, Spain | 89 | Prospective, multicenter, randomized for treatment comparison | Clinical improvement and parasitological control | | **x̃** 10.8 | 5/89 (5.6) | 11.7(PA)  6.7 (dAmB | 10/89 (11.2) | All patients: no prophylaxis | | 19/48 (39.6) | Univariate |
| Laguna, 1997 | | 1986 to 1994, Spain | 43 | Retrospective single center | Clinical improvement and parasitological control | | **μ** 9.9 ± 3.3 | 4/43 (9.3) | 29.4 | 14/43 (32.6) | 10 patients: no prophylaxis | | 5/10 (50) | Univariate |
|  |  |  |  |  |  |  | **μ** 10.4 ± 3.3 |  |  |  | 13 patients: pentamidine 4mg/kg once month | | 5/13 (38) |  |
| Fernandez, 1997 | | 1992 to January,1996 Spain | 31 | Retrospective single center | Clinical improvement | | **μ** 10.9 ± 5 | Not informed | 47.6 (PA)  3.3 (dAmB) | 26/31 (83.8) | 6 patients: no prophylaxis | | 4/6 (66.7) | Univariate |
|  |  |  |  |  |  |  |  |  |  |  | 17 patients: prophylaxis with drug not informed | | 3/17 (17.4) |  |
| Ribera, 1996 | | 1988 to 1995, Spain | 46 | Retrospective single center | Clinical improvement and parasitological control (some patients) | | Not informed | Not informed | Not informed | 3/46 (6.5) | 20 patients: no prophylaxis | | 13/20 (65) | Multivariate |
|  |  |  |  |  |  |  |  |  |  |  | 9 patients: Allopurinol 900mg/day | | 5/9 (6.6) |  |
|  |  |  |  |  |  |  |  |  |  |  | 17 patients: PA 850mg once a month | | 3/17 (17.6) |  |
| Montalban, 1989 | | 1983 to 1988, Spain | 16 | Retrospective multicenter | Not informed | | x̃ 12 (0.5-60) | Not informed | 12.5 | 6/16 (37.5) | All patients: no prophylaxis | | 5/14 (35.7) | Univariate |
