# Supplementary material for: Predictors of Visceral Leishmaniasis Relapse in HIV-Infected Patients: A Systematic Review
Source: PLoS Negl Trop Dis. 2011 Jun 7;5(6):e1153. doi: 10.1371/journal.pntd.0001153 (PMC3110161; doi:10.1371/journal.pntd.0001153)
Supplement: Table S2 — VL: Visceral leishmaniasis Parasitological confirmation: identification of Leishmania amastigotes by direct examination or by isolation of promastigotes in culture of tissue samples #Serology confirmation: Leishmania direct agglutination positive §Biologic confirmation: identification of Leishmania amastigotes by direct examination or by isolation of promastigotes in culture of tissue samples or Leishmania-specific PCR on peripheral blood/bone marrow dAmB: amphotericin B deoxycholate LAmB: liposomal amphotericin B LipAmB: amphotericin B lipid complex PA: Pentavalent antimonial compounds Hemo: transfusion route IDU: intravenous drug user HETERO: heterosexual contacts HOMO: men who have sex with men sexual: heterosexual or homosexual contacts SD: standard deviation IRQ: interquartile range ♯ if the information was available : median μ: mean. (DOC) [file pntd.0001153.s002.doc]

**Table S2. Visceral leishmaniasis relapse in HIV-1 infected patients**: characteristic of population

| *Reference* | *^x̃^Median or ^μ^mean age at primary VL diagnosis (range or SD) years* | *Gender male (%)* | *Diagnosis of AIDS prior VL (%)* | *Patients with each risk factor for HIV-1 transmission (%)* | *CD4+ baseline cell count ^x̃^median or ^μ^mean (range or SD cells/mL)* | *Patients with CD4+ < 200 cells/mL at primary VL diagnosis (%)* | *Visceral leishmaniasis diagnosis* | *Treatments of visceral leishmaniasis used (number of patients♯)* | *Patients under HAART at primary VL diagnosis (%)* | *Patients under HAART at VL relapse (%)* |
| --- | --- | --- | --- | --- | --- | --- | --- | --- | --- | --- |
| Ter Horst, 2008 | Without antiretroviral therapy: **x̃** 31.3(20-50) | 158 /161 (98.2) | Not informed | Not informed | Not informed | 71/ 161 (44,1) | Clinical signs and serology # or parasitological confirmation | PA 20mg/kg 30 days (323) or miltefosine 100mg/day 28 days | Not informed | Not informed |
|  | With antiretroviral therapy: **x̃** 33,5(18-60) | 176/195 (90.5) |  |  |  | 166/195 (85,6) |  |  |  |  |
| Bourgeois, 2008 | **x̃** 35 (30-51) | 22/27 (81.5) | 12/27 (44) | IDU: 12 (44.4) Homo: 6 (22.3) Hetero: 8 (29.6) Hemo: 1 (3.7) | **x̃** 51 (4 to 322) | 26/27 (96.3) | Clinical signs and biological^§^ confirmation | dAmB 0.8-1g cumulative amount or Ant (26) or PA 20mg/kg 30 days (1) | 12/27 (44) | Not informed |
| Molina, 2007 | **x̃** 36 (26-53) | 14/15 (93.3) | 7/12 (58.3) | IDU: 13 (86,7) Hetero 2 (13,3) | **x̃** 82 (4-210) | 12/12 (100) | Parasitological confirmation | LAmB 4mg/kg for 5 consecutive days and once per week thereafter for 5 more weeks (17) | 5/12 (42) | 8/9 (88.8) |
| Pasquau, 2005 | **x̃** 32 (IRQ 30–37) | 129/155 (83) | 94/155 (61) | IDU: 116 (75) Homo: 8 (5) Hetero: 20 (13) Unknown: 11 (7) | **x̃** 56 (20-120) | Not informed | Clinical signs and parasitological confirmation | PA 20mg/kg/d at least 21 days (139), dAmB - cumulative dose t least 1.5g (7), lipid formulations of amphotericin - cumulative dose at least 1.5g (5), pentamidine 4mg/kg/d 2 weeks (1) or fluconazole + allopurinol (2) | 24/155 (15) | 11/37 (30) |
| Mira, 2004 | **μ** 33 (24-57) | 19/21 (90) | 8/10 (80) | Not informed | Not informed | Not informed | Clinical signs and parasitological confirmation | PA 20mg/kg/day 28 days (13) or pentamidine 4mg/kg/day 28 days (2) or dAmB – 0.7mg/kg/d 28 days (5) or LAmB 2.5-4 mg/kg/d 10 days (9) or LipAmB 5mg/kg/day 14 days (5) | 0 | 19/21 (90.5) |
|  | **μ** 34 (26-37) | 10/10 (100) | 16/21 (76) |  |  |  |  |  | 0 | 10/10 (100) |
| López-Vélez, 2004 | **μ** 37+ 5 | 7/8 (87.5) | 7/8 (87.5) | Not informed | Not informed | Not informed | Clinical signs and parasitological confirmation | Not informed | Not informed | 8/9 (88.9) |
|  | **μ** 5+ 6 | 9/9 (100) | 4/9 (44.4) |  |  |  |  |  |  | 8/8 (100) |
| Fernandéz-Cotarelo, 2003 | **μ** 34.6 (range: 27–60) | 31/34 (91.2) | Not informed | IDU: 27 (77) Homo: 4 (11,2) Hetero: 2 (5.9) Unknown: 1 (2.9) | Not informed | Not informed | Clinical signs and parasitological confirmation | Not informed | 7/34 (20.6) | 10/13 (76.9) |
| Bossolasco, 2003 | **x̃** 37 (30-42) | 8/10 (80) | Not informed | IDU: 7 (70). Hetero: 2 (20) Homo: 1 (10) | **x̃** 42 (5-246) | 9/10 (90) | Clinical signs and parasitological confirmation | LAmB 3mg/kg/day on days 1-5 and once weekly thereafter between 17-66 days (10) | 4/10 (40) | 5/7 (71.4) |
| Casado, 2001 | **μ 3**4 (31-38) | 6/10 (60) | Not informed | IDU: 6 (60) Sexual: 4 (40) | **x̃** 70 (3–156) | 10/10 (100) | Clinical signs and parasitological confirmation | PA 20mg/kg 28 days or dAmB 0.7 mg/kg/day 28 days | 0 | 10 (100) |
| Pizzuto, 2001 | **x̃** 32 (27-45) | 8/10 (80) | 8/10 (80) | IDU: 8 (80) Hetero: 2 (20) | **x̃** 70 (4-190) | 10/10 (100) | Clinical signs and serology # or parasitological confirmation | PA (4) or LAmB (3) or dAmB (3) (“at standard doses”) | 5/10 (50) | 7/10 (70) |
| Pintado, 2001 | **μ** 33.2 + 8.2 | 64/80 (80) | 43/80 (53.7) | IDU: 63 (78.7) Homo: 6 (7.5) Hetero: 6 (7.5) Perinatal: 1 (1.3) Unknown: 2 (2.5) | **μ** 90 (3–470) | 61/70 (87.1) | Clinical signs and serology # or parasitological confirmation | PA 20 mg/kg/day - with a maximum daily dose of 850 mg) for 3–4 weeks (51) or dAmB 0.5–1 mg/day for 3–4 weeks (17) or Allopurinol + azoles compounds (4) | 2/73 (2.7) | Not informed |
| Berenguer, 2000 | **x̃** 37 (24-47) | 9/15 (60) | 7/15 (46.7) | IDU: 9 (60) Homo: 2 (13.3) Hetero: 3 (20) Unknown: 1 (6.7) | **x̃** 77 (3-215) | 13/14 (92.3) | Clinical signs and parasitological confirmation | PA 20 mg/kg per day for 28 days or LAmB total of 10 doses of 4 mg/kg per day given on days 1 to 5, 10, 17, 24, 31 and 38. | Not informed | 15/15 (100) |
| Villanueva, 2000 | **μ** 32.6 + 5,4 | 20/32 (62.5) | Not informed | Not informed | **x̃** 50 (1-200) with HAART **x̃** 69 (27-166) without HAART | Not informed | Clinical signs and parasitological confirmation | PA 20mg/kg/d 28 days (23) or LipAmB 3mg/kg/d 5-10 days (3) or LAmB 4mg/kg twice weekly 6 weeks (1) | Not informed | 5/5 (100) |
| Laguna, 1999 | **x̃** 32 (19–64) | 76/89 (85) | 56/89 (63) | IDU: 61 (69) Sexual: 16 (18) Others :12 (13) | **x̃** 20 (0-231) | Not informed | Clinical signs and parasitological confirmation | PA 20 mg/kg/day (44) or dAmB - 0.7 mg/kg per day (45), both for 28 days | 0 | 0 |
| Laguna, 1997 | Not informed | 42/43 (98) | 29/43 (67) | IDU: 35 (81) Sexual: 7 (16%) Others :1 (2) | **μ** 10 ±3.3 | Not informed | Clinical signs and parasitological confirmation | PA low dose:< 20 mg/kg/day 21 days (17) or PA high dose: ≥20 mg at least 28 days (29) or dAmB (1) or LAmB (4) | 0 | 0 |
| Fernandez, 1997 | **μ** 34,3+ 5,1 | 30/31 (96.7) | 18/31 (58.1) | IDU :21 (67.7) Homo: 6 (19.4) Hetero: 4 (12.9) | **μ** 36,9 ± 27,9 | 31/31 (100) | Clinical signs and parasitological confirmation | PA 20 mg/kg/d - maximum 850mg/d 21 days (21) or dAmB 1-1.5 g cumulative amount - 0.5 mg/kg per day (20) | 0 | 0 |
| Ribera, 1996 | No prophylaxis **x̃** 27 (20-64) | Not informed | 9/20 (45) | IDU: 9 (45) | **x̃** 35 (2-125) | Not informed | Clinical signs and parasitological confirmation | PA 850mg/day 21 days or dAmB (52) | 0 | 0 |
|  | Allopurinol prophylaxis **x̃** 28 (22-29) |  | 6/9 (67) | IDU: 5 (55) | **x̃** 11 (2-42) |  |  |  |  |  |
|  | PA prophylaxis **x̃** 29 (21-36) |  | 3/17 (18) | IDU: 13 (76) | **x̃** 34 (8-268) |  |  |  |  |  |
| Montalban, 1989 | Not informed | 14/16 (87.5) | 5/16 (31) | IDU: 15 (94) Homo:1 (6) | Not informed | Not informed | Clinical signs and parasitological confirmation | PA (16) | 0 | 0 |
