# Supplementary material for: Predictors of Visceral Leishmaniasis Relapse in HIV-Infected Patients: A Systematic Review
Source: PLoS Negl Trop Dis. 2011 Jun 7;5(6):e1153. doi: 10.1371/journal.pntd.0001153 (PMC3110161; doi:10.1371/journal.pntd.0001153)
Supplement: Table S3 — Yes: positive association No: negative association VL: Visceral leishmaniasis HAART: highly active antiretroviral therapy HVC: hepatitis C virus * multivariate analysis. (DOC) [file pntd.0001153.s003.doc]

**Table S3. Visceral leishmaniasis relapse in HIV-1 infected patients: variables investigated**

| *Variables* | *Ter Horst, 2008* | *Bourgeois, 2008* | *Molina, 2007* | *Pasquau, 2005* | *Mira, 2004* | *López-Vélez, 2004* | *Fernandéz-Cotarelo, 2003* | *Bossolasco, 2003* | *Casado, 2001* | *Pizzuto, 2001* | *Pintado, 2001* | *Berenguer, 2000* | *Villanueva, 2000* | *Laguna, 1999* | *Laguna, 1997* | *Fernandez, 1997* | *Ribera, 1996* | *Montalban, 1989* |
| --- | --- | --- | --- | --- | --- | --- | --- | --- | --- | --- | --- | --- | --- | --- | --- | --- | --- | --- |
| Tuberculosis co-infection | Yes * |  |  |  |  |  |  |  |  |  |  |  |  |  |  |  |  |  |
| VL previous | Yes * |  |  |  |  |  |  |  |  |  |  | No | Yes |  |  |  | Yes * |  |
| Age |  | No |  | No |  |  |  | No |  | No |  | No |  |  |  |  |  |  |
| Female gender |  | No |  | Yes * | No |  |  | No |  | No |  | No |  |  |  |  |  |  |
| Intravenous drug use |  | No | No | No | No |  |  | No |  | No |  | No |  |  |  |  |  | No |
| Homosexual contact |  | No |  |  |  |  |  |  |  |  |  | No |  |  |  |  |  |  |
| AIDS previous to VL |  | No | No | No | No |  |  |  |  | No | No * | No |  |  |  |  | No * | No |
| PCR HIV load at baseline |  | No | No |  | No |  |  | No | No | No |  |  | No |  |  |  |  |  |
| PCR HIV load at follow-up |  |  |  |  | Yes |  |  | No |  |  |  | No | No |  |  |  |  |  |
| Antiretroviral therapy use at VL diagnosis | Yes * |  |  |  |  |  |  |  |  |  |  |  |  |  |  |  |  |  |
| Antiretroviral therapy use at follow-up |  |  |  |  |  |  |  |  |  |  | No * |  |  |  | No |  |  |  |
| HAART use at VL diagnosis |  | Yes | No |  |  |  | No |  |  | No |  |  |  |  |  |  |  |  |
| HAART use at follow-up |  |  |  | No |  |  | No | No |  | No |  |  | No |  |  |  |  |  |
| HAART compliance |  |  |  |  | No |  |  |  |  |  |  |  |  |  |  |  |  |  |
| CD4+ count at VL diagnosis |  |  | No | No | No |  |  | No | No | No | No * | No |  |  |  |  | No * |  |
| CD4+ count < 100 cell/mL at VL diagnosis | Yes * | Yes * |  |  |  |  |  |  |  |  |  |  | No |  |  |  |  |  |
| CD4+ count < 200 cell/mL at VL diagnosis |  |  | No |  |  |  |  |  |  |  |  |  |  |  |  |  |  |  |
| CD4+ count at follow-up |  | Yes | Yes |  | Yes |  |  | Yes | No |  |  | Yes | No |  |  |  |  |  |
| HCV co-infection |  | Yes * |  |  |  |  |  |  |  |  |  |  |  |  |  |  |  |  |
| Sexual transmission |  |  | No |  |  |  |  |  |  |  |  |  |  |  |  |  |  |  |
| Completed therapy for VL |  |  |  | Yes * |  |  |  |  |  |  |  |  |  |  |  |  |  |  |
| Specific anti-*Leishmania* treatment given |  |  |  |  |  |  |  |  |  |  |  | No | No | No |  |  |  |  |
| Secondary prophylaxis use (non specificated regimen) |  |  |  | Yes * |  |  |  | No |  |  |  |  |  |  |  | Yes |  |  |
| Antimonial secondary prophylaxis |  |  |  |  |  |  |  |  |  |  | Yes * |  |  |  |  |  | Yes |  |
| Liposomal amphotericin secondary prophylaxis |  |  |  |  |  |  |  |  |  |  | Yes * |  |  |  |  |  |  |  |
| Pentamidine secondary prophylaxis |  |  |  |  |  |  |  |  |  |  |  |  |  |  | No |  |  |  |
| Amphotericin complex lipid secondary prophylaxis |  |  |  |  |  | No * |  |  |  |  |  |  |  |  |  |  |  |  |
| Clinical findings (hepatomegaly, splenomegaly, anemia, leucopenia, thrombocytopenia) |  |  |  | No |  |  |  |  |  |  |  |  |  |  |  |  |  |  |
| Time from de VL diagnosis to the introduction of protease inhibitors therapy |  |  |  |  |  |  |  |  | No |  |  |  |  |  |  |  |  |  |
| Anti-*Leishmania* antibody positive |  |  |  |  |  |  |  | No |  | No | No * |  |  |  |  |  | No |  |
